# ‘I Don't Like Uncertainty, I Like to Know’: How and why uveal melanoma patients consent to life expectancy prognostication

**DOI:** 10.1111/hex.13490

**Published:** 2022-04-26

**Authors:** Stephen L. Brown, Peter L. Fisher, Andrew Morgan, Cari Davies, Yasmin Olabi, Laura Hope‐Stone, Heinrich Heimann, Rumana Hussain, Mary Gemma Cherry

**Affiliations:** ^1^ School of Psychology University of Plymouth Plymouth UK; ^2^ Department of Psychological Sciences University of Liverpool Liverpool UK; ^3^ Liverpool Ocular Oncology Centre Liverpool University Hospitals NHS Foundation Trust Liverpool UK

**Keywords:** medical ethics, patient decision‐making, prognostication, qualitative, uveal melanoma

## Abstract

**Background:**

Technological advances have led to cancer prognostication that is increasingly accurate but often unalterable. However, a reliable prognosis of limited life expectancy can cause psychological distress. People should carefully consider offers of prognostication, but little is known about how and why they decide on prognostication. Using uveal melanoma (UM) patients, we aimed to identify (i) how and why do people with UM decide to accept prognostication and (ii) alignment and divergence of their decision‐making from conceptualizations of a ‘well‐considered’ decision.

**Methods:**

UM provides a paradigm to elucidate clinical and ethical perspectives on prognostication, because prognostication is reliable but prognoses are largely nonameliorable. We used qualitative methods to examine how and why 20 UM people with UM chose prognostication. We compared findings to a template of ‘well‐considered’ decision‐making, where ‘well‐considered’ decisions involve consideration of all likely outcomes.

**Results:**

Participants wanted prognostication to reduce future worry about uncertain life expectancy. They spontaneously spoke of hoping for a good prognosis when making their decisions, but largely did not consider the 50% possibility of a poor prognosis. When pressed, they argued that a poor outcome at least brings certainty.

**Conclusions:**

While respecting decisions as valid expressions of participants' wishes, we are concerned that they did not explicitly consider the realistic possibility of a poor outcome and how this would affect them. Thus, it is difficult to see their decisions as ‘well‐considered’. We propose that nondirective preference exploration techniques could help people to consider the possibility of a poor outcome.

**Patient or Public Contribution:**

This paper is a direct response to a patient‐identified and defined problem that arose in therapeutic and conversational discourse. The research was informed by the responses of patient participants, as we used the material from interviews to dynamically shape the interview guide. Thus, participants' ideas drove the analysis and shaped the interviews to come.

## 1 INTRODUCTION

Prognostication is the process of forecasting and communicating future clinical outcomes.^1^ Benefits are informed treatment decision‐making for clinicians and patients, and greater certainty and a platform for life planning for patients. Although a poor prognosis brings the risk of psychological distress,^2^ prognostication is desired by many.^3^

Historically, prognostic estimates have often been uncertain,^4^ but recent technical developments, particularly in genetics, are driving a trend toward greater accuracy. For example, cytogenetic analysis of tumour cells enables reliable life expectancy estimates in several cancers.^5^, ^6^, ^7^, ^8^, ^9^ However, advances in prediction are not always accompanied by treatment development, and poor prognoses sometimes cannot be effectively ameliorated.^1^ Receipt of accurate but irreversible poor prognoses can cause potentially severe psychological distress.^10^, ^11^, ^12^, ^13^ This subtly changes the focus of prognostic decision‐making. With more accurate predictions, but few ameliorative treatments, people need to make a finely balanced decision as to whether they prefer certainty or uncertainty, and whether to risk the psychological burden of a poor prognosis.^10^ For practitioners, heightened risk of distress sharpens existing ethical dilemmas about how to offer prognostic testing and communicate its results and inform and support patients' decision‐making.^1^

Kleinman^14^ proposes that ethical theory can be inductively shaped by critical observation of contemporary practice. Huntington's disease (HD) has influenced ethical positions on accurate but nonameliorable prognostication, but transferability to cancer is uncertain due to distinctive HD illness course, patient age and heritability attributes.^15^ Uveal melanoma (UM), an eye cancer, provides a cancer paradigm where prognostic advances are not matched by contingent treatment advances. UM is treatable, and cytogenetic analysis of tumour tissue provides reliable predictions of life expectancy. Receiving a poor prognosis predicts a shortened life expectancy that generally cannot be remediated by current treatments.^16^ People with UM should carefully consider the benefits and costs of receiving a prognosis. To identify how to best support decision‐making, we aimed to better understand how and why people decide to accept offers of prognostic testing.

### 1.1 Prognostication in UM

Research has focussed on prognostic disclosure, patient awareness, postprognosis decision‐making and patient distress,^17^, ^18^, ^19^, ^20^ rather than patients' decisions to undergo testing and to receive a prognosis. Qualitative studies suggest that people want prognoses to inform procedural choices and to resolve uncertainty,^18^, ^21^, ^22^ often after a medical or family recommendation.^23^ Patients also see prognostication as communication with healthcare professionals that, done well, is imbued with caring and emotional support that has value to patients.^17^, ^23^ Quantitative predictors of acceptance include familial risk, worry, perceived vulnerability, low cognitive avoidance, dispositional optimism and expectation of unambiguous results.^24^, ^25^, ^26^, ^27^, ^28^

Primary UM tumours are usually treated successfully, but 40%–50% of treated people later die of metastatic melanoma.^29^, ^30^ Metastatic risk is predicted by cancer cell morphology and a mutation involving deletion of one of the pair of chromosome 3 alleles. People with UM are offered prognostic testing^a^ with results provided by postage within 6 weeks. About 60% choose testing and almost all elect to receive results. Some must decide within 1 week of diagnosis, while others have up to 10 weeks. Test failure is about 5%. The test shows good all‐cause mortality prediction with C‐statistics 0.79–0.80,^31^ and 0.81 sensitivity and 0.72 specificity predicting metastatic melanoma^32^ Test failure is about 5%. Risks are vision loss and tumour seeding. Those with poor or no prognoses are referred to an oncologist and offered screening. Treatments cannot significantly extend or improve life to a significant degree in most people.^16^, ^33^

At a population level, two large 2–5‐year prospective studies show that a poor prognosis is related to moderately but consistently elevated anxiety, depression and worry about cancer recurrence compared to a good prognosis or no prognosis.^10^, ^34^ Outcomes of a good prognosis do not differ from no prognosis. Distress in the poor prognosis group may not significantly exceed healthy age and gender‐matched population means.^10^, ^34^ Population characteristics mask individual variability. Some experience uncertainty and regret after their decision to undertake a prognostic test, while others are satisfied with their decisions irrespective of prognostic outcome.^3^, ^35^

Although treatments for metastatic melanoma have limited medical benefits, patients can feel supported and cared for within screening programmes.^35^ Similarly, some may feel that providing tumour samples provides benefits to others by supporting research.^21^ Nonetheless, in the absence of mortality or morbidity benefits of treatment, decision‐making approaches equipoise; a preference‐based choice where the risk of distress and the risks of the procedure should be considered against benefits.^19^, ^22^ The substantial risks underscore the importance of making ‘well‐considered’ decisions; defined as decisions likely to generate outcomes that reflect and further an individual's values and priorities.^36^, ^37^, ^38^, ^39^, ^40^, ^41^ ‘Well‐considered’ decisions are often defined in process terms because individual decision outcomes are frequently affected by factors unpredictable at the time of decision‐making. A consensus among researchers is that decision‐makers should understand and consider the risks and benefits of choices, then reach a decision that logically integrates these considerations.^39^, ^40^, ^41^

Decision‐making in cancer can be imbued with constraints. Disorientated and helpless patients often struggle to process complex technical information,^18^, ^20^, ^37^, ^38^ arguably reducing the capacity for effective self‐determination.^37^, ^38^ From a relational autonomy perspective, several authors^20^, ^37^, ^39^ argue that practitioners ought to support and, where necessary, assist people to make well‐considered decisions. In one study of UM study, most participants, emotionally overwhelmed by their diagnosis and decision complexity, did not make considered decisions. They chose prognostication because they misinterpreted clinicians' offers of prognostication as recommendations for it.^42^

### 1.2 Current study

The nature of practitioner support will depend upon identifying and targeting specific reasons why decisions may or may not be well‐considered. However, decision‐making about undergoing testing and receiving a prognosis in UM is not well understood.^10^ We used a sample of UM patients who chose prognostication because the risk of distress becomes elevated only after a poor prognosis. Our research questions were: (i) how and why did people with UM decide to accept prognostication? and (ii) to what extent did their decision‐making align with or diverge from current conceptualizations of a ‘well‐considered’ decision?^36^, ^37^, ^38^, ^39^, ^40^, ^41^

## 2 METHODS

### 2.1 Participants

This project was funded by the Liverpool University Hospitals NHS Foundation Trust Charitable funds: A0982/CF Eye Tumour Research Fund. The study received ethical approval from the North West Greater Manchester Research Ethics Committee: 17/NW/0542. We invited people with UM aged 18 years or above, treated at the Liverpool Ocular Oncology Centre (LOOC) following a clinical diagnosis of UM, who had been offered a prognostic biopsy and initially chose it. Upon the return of a consent to contact form by mail, the researchers (D. F. and A. M.) contacted interested people with UM by telephone and provided further details about the study. For those willing to participate and who chose prognostication, the date and time were arranged for the interview to be conducted. Consent was given immediately before interview through a returned form and audio‐recorded verbal consentrview. The sample size was largely determined by a low case rate during the study window rather than theoretical saturation. A post hoc examination of the initial themes indicated some saturation, as the last three participants did not suggest new themes, but saturation cannot be assumed.

### 2.2 Procedure

Interviews were conducted after their biopsy but before the results were available (except for P05 who received results immediately before interview). Three participants with UM changed their initial decision and declined the prognostic biopsy; their interviews were scheduled to take place after the prognostic biopsy would have been taken. All interviews were by telephone. The interviewers (D. F. and A. M.) used a semi‐structured interview schedule, using open questions, prompts and reflection to facilitate participants' talk. Closed questions probed specific points. Pacing, sequencing and length of interviews were set by participants. Interviewers followed a topic list but also pursued what they considered relevant participant‐generated ideas absent from the list. Interviews were read by S. L. B. after the interview as quality control to ensure that interviewers pursued topics and research goals. Initial interview topics are presented in Table 1. Further elements were added as the interviews unfolded. Interviews were audio‐recorded, transcribed verbatim and pseudo‐anonymized.

**Table 1:** Initial interview topic guide

*Background* How did you hear about the test/biopsy? At what point did you first consider the prognostic test/biopsy offer? What do you recall of the prognostic offer? What sort of information did you seek, to help you make that decision? *Decision‐making* What were your initial thoughts about prognostic testing? To what extent did you feel that you understood the decision you were asked to make? Could you please describe how you made the decision to have/not have test/biopsy? What sort of issues did you consider when you were deciding whether you wanted the test/biopsy? Of these, which issues were important to you? How did the role of worry/emotions/risk shape up your decision? *Decision context* Which clinical staff did you talk to about your decision? How did each staff member respond to you? Who raised the prospect of you having a test/biopsy? What did you want to get out of the discussions with your family/doctor/nurse/counsellor? Did (family/doctor/nurse/counsellor) speak to you about the risks associated with your decision? Could you describe how talking to clinical staff or family influenced your decision? *Decision satisfaction* How do you feel about your decision now? When was it clear to you that you wanted this? What were your thoughts at that time? Does the participant have any questions or any concerns about anything that has been discussed?

### 2.3 Analysis

Our philosophical orientation towards the analysis corresponded to subtle realism; the position that the phenomena being investigated stand independently of the investigation, but its interpretation by investigators is inherently fallible.^43^ We took a pragmatic approach where we sought what Levitt et al.^44^ describes as ‘methodological integrity’; the pluralistic use of analytic techniques to ensure fidelity and utility. Fidelity is the quality of our interpretation of the phenomenon. Using template analysis,^45^ we used a database in Microsoft Word to document and code text.^46^ Descriptive themes were developed from the codes. Theoretical development proceeded using a constant comparative ‘cycling’,^47^ moving forwards and backwards between new text and previous cases to test the fidelity of interpretations against that data. The utility is linking the analysis to research goals. The interview guide ensured the collection of data that addressed research questions. The development of theory as to how and why the obtained themes aligned and diverged from ‘well‐considered’ decisions was supported by a comparison of the final themes to the normative conception of ‘well‐considered’ decision‐making.^23^, ^24^, ^25^, ^26^, ^27^

The analysis was conducted parallel to interviewing, enabling the developing analysis to inform subsequent iterations of the interview guide. C. D. and Y. O. created an initial template of codes and a descriptive thematic framework. Further theme development and refinement was conducted by S. L. B. and C. D., who emphasized and refined themes in the light of research questions and compared the emerging analysis to the conceptualization of a ‘well‐considered’ decision. This analysis was periodically taken to the wider group and modified until agreed upon by the majority. Disagreements between two readers at the text level were addressed by discussion. If not agreed, they were brought to the group for majority agreement. Disagreements at the interpretive level were resolved within the group by majority agreement.

All group members read at least some interviews, S. L. B., C. D. and Y. O. read all of the interviews. The research team brought an explicitly psychological perspective to the analysis. S. L. B. and L. H.‐S. are health psychologists with experience in practice and research in decision making in UM. S. L. B. has conducted decision‐making research across a range of physical health settings. L. H.‐S. is the psychological lead for LOOC with responsibility for assisting patients to make prognostication decisions. P. F., G. C. and A. M. are clinical psychologists with experience in physical health settings. C. D. is an assistant psychologist and research assistant working in psycho‐oncology and Y. O. a clinical psychology trainee. S. L. B. and L. H.‐S. both have experience in leading and publishing qualitative research projects. H. H. and R. H. are surgeons who regularly offer prognostication to patients were available to clarify medical issues and advise on how prognostication was offered but did not participate in the analysis.

Key findings are illustrated by italicized quotes attributed to individuals (participant numbers in parenthesis), with ellipses (…) indicating omitted text and explanatory comments in square brackets.

## 3 RESULTS

Participants were 11 women and 9 men, with a median age of 56.5 years (range: 26–75 years). Median time between diagnosis and planned biopsy was 3.14 weeks (range: 1.14–11.86 weeks). Interviews were a median 36 min (range: 23–68 min). Table 2 shows clinical and sociodemographic characteristics.

**Table 2:** Participant characteristics

PT ID	Age band	Sex	Marital status	Emp status	Education	Treatment	Biopsy Y/N	HADS‐Anxiety	HADS‐Depression	HADS‐Total
005	60–69	F	Married	P/T	GCSE	Plaque	Y	12	4	16
007	60–69	F	Civ Part	P/T	PG	Plaque	N	10	11	21
008	50–59	F	Married	Unemployed	A' levels	Proton beam	Y	5	6	11
009	40–49	M	Married	F/T	UG	Plaque	Y	2	1	3
011	40–49	F	Single	F/T	GSCE	Proton beam	Y	12	1	13
014	40–49	F	Married	F/T	UG	Plaque	N	0	1	1
019	50–59	M	Partner	P/T	GCSE	Plaque	Y	11	1	12
021	60–69	M	Married	F/T	GCSE	Plaque	Y	2	1	3
025	60–69	F	Married	F/T	PG	Proton beam	N	6	2	8
029	50–59	M	Married	F/T	Diploma/Voc	Plaque	Y	5	4	9
031	50–59	F	Divorced	F/T	GCSE	Plaque	Y	16	11	27
046	50–59	M	Married	F/T	GCSE	Plaque	Y	2	3	5
047	50–59	F	Partner	Retired	UG	Plaque	Y	6	2	8
049	50–59	M	Married	F/T	A'Level	Plaque	Y	6	1	7
050	30–39	F	Single	P/T	UG	Proton beam	Y	14	6	20
059	70–79	F	Married	Retired	UG	Plaque	Y	7	5	12
074	20–29	F	Single	F/T	UG	Proton beam	Y	3	2	5
089	70–79	M	Married	Retired	Diploma/Voc	Plaque	Y	3	2	5
092	50–59	M	Single	F/T	A'Level	Plaque	Y	8	7	15
095	70–79	M	Married	Retired	Diploma/Voc	Plaque	Y	7	1	8

Abbreviations: A levels, university qualifying qualification; Diploma/Voc, vocational qualification; F/T, full time; GCSE, secondary school qualification; P/T, part time, UG, undergraduate degree.

### 3.1 Summary of findings

Participants largely understood their choice and the emotional consequences of a poor prognosis. Rather than considering multiple outcomes and their implications, a single consideration dominated decision‐making; participants intensely feared worry associated with uncertainty and wanted to reduce it. Most made ‘gut’ decisions to accept testing. Participants hoped for and some expected a good prognosis, but none reported taking the possibility of a poor prognosis into consideration. Three participants later declined prognostication because procedural risks deterred them.

### 3.2 Background to the decision

#### 3.2.1 The offer of prognostication was not always initially understood, but participants were motivated to understand it

About half of our participants (Ps. 07, 08, 09, 11, 29, 47, 59, 89, 92, 95) struggled to follow the consultation where the offer of prognostication was made. Common experiences were shock, disorientation and numbness. Most felt bombarded with information they found difficult to understand and integrate: ‘and there was such a lot of information to take in. I know I came back with loads of leaflets and forms that I signed and things like that. So all that happens really quickly I have to say, and in the heat and the aftermath of a diagnosis’ (P07).

Yet, most applied themselves to understanding the prognostic offer. They generally eschewed external, particularly online, resources. Instead, they consulted unit resources such as written information provided by the ocular oncology centre: ‘I mean to be honest the booklet that the hospital provided was probably one of the most comprehensive things that we got……I think that was, that's where it was helpful in that it wasn't saying you must have this done, do you know what I mean’ (P14). Healthcare professionals were also trusted: ‘we had a discussion through it [with nurses] and got all the leaflets which I then brought home and, it was very good, it was very, for me it was err encouraging, positive supportive all that you know it was very good you know’ (P21).

#### 3.2.2 Participants understood the purpose of prognostication

Almost all participants grasped the central idea of prognostication, that a biopsy would be conducted to determine the likelihood that cancer would spread and thus endanger life and that they would receive a clear prediction. They were able to differentiate this from the diagnostic component of the biopsy (confirming the existence of melanoma) ‘I realised then it was more to find out how to make a prognosis based on the cells that are removed, you know the type of cells they are rather than just confirming it's a melanoma’ (P05).

Participants understood the consequences of a poor prognosis; ‘So I was more worrying about whether it was going to spread. That was my main thought, was if it's going to spread and if I'm going to die, and the way to find that out is the biopsy’ (P50), and that it could be emotionally difficult: ‘I think one does think ahead and think, Well it's all very well knowing if its high risk or low risk but of course err you know if it's not low risk you're going to feel like poo’ (P07).

Less widely understood was that treatment for metastatic UM rarely prolongs life. Clinicians state this to participants. Eight (Ps 19, 21, 29, 31, 46, 50, 74, 95) spoke specifically of treatments: ‘having spoken afterwards and thought about it and spoke to [ocular oncology nurse] you know I need to be aware of where I am and what I've got and if I have got, if it is going to my liver I need to be able to have the best possible treatment and having the biopsy done that will give me the best possible’ (P31). P95 spoke of a cure: ‘I can have treatment for it, erm, and we know its aggressive and if it's sort of in the early stages, hopefully we can have treatment for it and maybe, I don't know, cure it or delay it or something like that’.

### 3.3 Why participants wanted a prognosis

#### 3.3.1 Participants anticipated and feared worry about uncertainty

All participants' dominant concern was living with worry for the rest of their lives; ‘because then the worry worrying all the time would take over my life…I can't live like that’ (P5). Several appeared to infer their futures from present feelings, as was indicated by their use of the present tense to speak about emotion, ‘Because if I hadn't had the biopsy… I mean, I could have a lump on my big toe at the minute and it would be cancer, that's the way I feel. I've gone paranoid’ (P31).

Participants were particularly disturbed by the unpredictability and inescapability of intrusive thoughts: ‘The impending doom…. school bully waiting at the school gates. We've all had that feeling erm where he's going to get you at the school gates on the way home and you forget about it and then suddenly remember it and you think, So whatever you're doing, whether you're reading, whether you're walking, whether you're watching a movie, you can be enjoying it one minute and then you think, ‘God, oh yes, that's the state I'm in at the moment’ (P92).

#### 3.3.2 Participants wanted to eliminate uncertainty

Thirteen participants (Ps 5, 7, 8, 11, 14, 21, 29, 31, 47, 50, 74, 92, 95) stated that a prognosis would assuage their fears. ‘That [risk of spread] obviously puts you in a kind of a situation where there is a little bit of uncertainty I suppose and obviously the way in which that uncertainty is clarified is undertaking a biopsy on the melanoma’ (P09). Most expressed their ‘need’ for prognostication *forcefully* and emotionally: ‘that's why I think again I've gone for the biopsy because I need to know, I need to know’ (P31); ‘having that sat over you all of the time knowing that what you know was it high or low risk. I'm not sure I could live, you know, live like that. [Prognostication would] remove the dread and fear attached to uncertainty’ (P29).

A prognosis represented a ‘tangible’ (P08) base from which to resume lives put on hold by uncertainty: ‘The more knowledgeable you are about your own condition then the better chance you have of you know living with it successfully, enjoying your life etc. and sort of being able to you know carry on with things as normally as you can kind of thing really’ (P47).

Six (Ps 5, 8, 9, 11, 29, 95) mentioned that their wishes to reduce uncertainty were linked to longstanding preferences for coping with adversity ‘I think it's part of what my role is in [that] it's my working role as well, you know. I'm an analyst by trade so I don't like uncertainty. I like to know, you know, as much information as I can possibly have and then you can obviously undertake a review of that and see what the options are’ (P09); ‘I just, yeah, because it suits my personality to know and deal than not know’ (P08). Others mentioned active approaches to coping: ‘so to me that's [declining the test] a bit like, not cowardice, but like burying your head a bit, you think at least you're armed with the information then you can deal with it’ (P05).

### 3.4 How participants decided

#### 3.4.1 ‘Gut decisions’ and ‘right decisions’

Most participants eschewed extensive deliberation before accepting prognostication: ‘I just made a decision that I wanted to know… and I on purpose didn't even research too much into er, you know the actual er nature of the cancer’ (P8). P29 described the decision as a ‘gut’ decision: ‘I do act upon erm my gut sort of gut feeling instinct if you like, I'm a big believer in that… just going off experience it's normally right’. These participants were certain about their preferences. P19 described the decision as a ‘no brainer’—obviously the right thing for her to do: ‘It was it was it was just one of those things. I wanted it done and that was it, I didn't even really think about it’.

A smaller number of participants wanted to take their time to make a considered, ‘right’, decision. They adopted one of two approaches. The first group initially preferred a prognosis, but opted for further research and consultation to ‘test’ their preference. ‘I just said, “Oh, I've been thinking about it and I really think I should have the biopsy”’ (P11). These participants were open to health professionals' views: ‘I was willing to change my decision if, when I got the information from [ocular nurse] or the doctor at [cancer hospital], if they'd said anything that would have made me change my mind I would have done it’ (P25).

Some of this smaller group embarked on their considerations from a state of apparent equipoise: ‘I was completely out of my comfort zone and then you get that news and you're it was a lot to take in, a lot…so I didn't, I didn't just jump in it feet first, I wanted to read it, you know. I said, “I just need time to read through it and see what it says”’ (P11).

Nine participants spoke to family members, but they did not always open the decision to them, seeking confirmation and support for decisions already made: ‘Erm so really it was just affirming that with everybody around me, everybody was saying, “Well, I would do the same. I would do the same” erm and that's really how I got to that point where I thought, “Well, that's probably erm the right thing for me”’ (P65). Participants were also keen to protect the family *from* the decision: ‘I kind of wanted to protect my wife. Erm, my wife's not been the best of health herself. She suffers from quite bad anxiety… so I kind of didn't really tell my wife my worst fears, because I didn't want her worrying in case there was nothing to worry about’ (P49).

#### 3.4.2 Participants hoped for and expected a good prognosis

Nine participants (Ps 11, 14, 25, 29, 31, 47, 49, 92, 95) emphasized the advantages of a good prognosis: ‘My decision to have it done is my children, so I can say to them, “Brilliant news, you know. It's low risk and it's not going to go anywhere else at the moment”’ (P31).

P31's quote suggests that she moved beyond hope, towards some level of expectation that her prognosis might be good. This expectation was shared by others. But the reasonings that they used to justify their *optimism* were often not logical: ‘I'm always optimistic so, erm, you know, I feel good in myself. None of my, none of my body has changed, you know. I'm still as healthy as I was. I'm still going. I haven't lost weight. My toiletry habits are the same and just things that, you know, things that you were told you might need to look out for. Nothing's changed so I am quite confident that the results are going to be ok’ (P49).

#### 3.4.3 How participants considered the possibility of a poor prognosis

Participants were aware that a poor prognosis could be emotionally difficult (described in Section 1.2), but took this possibility into consideration only when it formed a favourable argument for prognostication. Some participants noted, reasonably, that a poor prognosis may entitle them to future developments in treatments: ‘I assume people that are younger, like myself, would rather know because we've got far more years for it to kind of resurface or more treatment to happen and things along those lines’ (P74). Other participants saw treatments not specifically as a means of extending life, but in terms of being cared for or ‘being in good hands’ (P21) of their healthcare team: ‘I know that you know the great strides that we are doing in general treatment not just cancers in all sorts, in all sorts of diseases, that we are just striving forward and I just have confidence’ (P21).

Others (Ps. 49, 50, 59) felt that a poor prognosis would enable them to prepare for the possibility of early death: ‘I need to know, you know. I've got a wife and I've got children and I'd rather know and then I could prepare financially and things you know. “Cause my wife and I were thinking of moving house before this happened”’ (P49).

Participants' recalled thinking about reasons favouring accepting the test; the desirability of a good prognosis or practical incentives to learn of a poor prognosis. In contrast, none recalled thinking about a poor prognosis as a disincentive to prognostication. ‘I didn't give it [a poor prognosis] any consideration. I just felt that whatever it would be it would, I could more easily cope knowing than not knowing’ (P08).

During the study, we started specifically asking about a poor prognosis. Participants did not report considering it as a disincentive. Several produced what appeared to be post hoc reasoning. Ten argued that it is a risk worth bearing for certainty (Ps 5, 7, 8, 9, 19, 21, 29, 74, 92, 95), with some viewing it almost as a positive event because it allowed the possibility of *coping* which they felt was denied to them by uncertainty: ‘If you don't get it done you're going to be living in fear. It's like, if you do get it done and it is bad news you can fight it. You can deal with that’ (P92). Several (Ps 9, 29, 31, 49, 50, 95, 74) preferred a poor prognosis to not knowing because they could plan their future for themselves or their families: ‘Interviewer: “Would you rather—this is an abstract question—would you rather have bad news for certain or not know at all?” Patient: “Erm, I think it would have to be bad news for certain because then I could manage my future better I think”’ (P29).

### 3.5 Three participants changed their minds and declined prognostication after reviewing their decisions

Three participants (Ps 7, 14 and 25) initially wanted a prognosis, but later decided against it after further *consideration* due to the risk of damaged vision and tumour seeding: ‘It was the risk factors. It is mainly the risk factors associated with the biopsy that's making me decide not to have it’ (P25). P14 spoke with a doctor (friend) which changed her mind: ‘Initially I would of just had it done but it was only sort of once I'd had this chat that's when sort of doubts entered and that's then when I started looking at it properly because I think you just get into this system of right you're going to have this this and this done so it was only when he sort of gave the sort of pros and cons of having it done that I then thought, “Right, ok, we'll look into this further”’ (P14).

## 4 DISCUSSION

Participants largely understood the offer of a prognosis and its consequences. They chose it because they otherwise dreaded a future of worry over uncertainty.^17^, ^21^ Participants were generally confident in their choices, did not require assistance to make their decision^22^, ^23^ and were aware of potential consequences. Decisions also reflected reasonable hopes that treatments may be found, pragmatic motivations and expressed participants' self‐perceptions as people who *address* problems directly. These reasons are commonly cited by people seeking prognosis.^20^, ^21^, ^23^ Participants' fears of being unable to tolerate uncertainty are theoretically reasonable,^48^ and they ‘owned’ decisions in the very real sense that they wanted to make the decision and in doing so pursued a goal that they valued.

While the reasons *why* participants opted for a prognosis seem clear. Some concerns seem warranted over *how* they made their decisions. First, although informed, a small number of participants failed to fully understand the decision task, particularly those expecting that a prognosis would lead to effective treatment. Where participants misunderstand the decisions that they are making or hold objectively incorrect beliefs, their consent cannot be regarded as fully understanding the decision.^42^ Such misunderstandings would need to be identified and addressed.

Second, we defined a ‘well‐considered’ decision as one where people consider relevant outcomes and try to logically integrate them into decision‐making.^13^, ^14^ A ‘well considered’ decision process to undergo testing and to receive a prognosis would involve thinking about at least three outcomes; the consequences of a good prognosis, a bad prognosis or remaining uncertain. Participants reasonably wanted certainty that would allow them to move forward with planning their lives, and some anticipated value even in certainties afforded by a poor prognosis.^17^ Others chose not to have testing due to the risks of biopsy, although this consideration followed an initial decision to have a prognosis. However, similar to other studies,^49^, ^50^ participants' decisions were dominated by the single, highly salient, goal of reducing anticipated distress associated with uncertainty. This goal was associated with a bias towards consideration of reasons for rather than against a prognosis. We infer bias in comparing the lengths to which participants thought about the possibility of a good prognosis, and also a poor prognosis mainly when it favoured existing preferences for a prognosis, with none spontaneously reporting thinking about a poor prognosis in the context of a deterrent. When asked, participants explained a possible poor prognosis using the same terms as they used to decide upon receiving a prognosis in the first place; that a certain poor prognosis would be at least preferable to uncertainty.

In short, tensions exist between giving individuals the autonomy to make the decisions they want in the ways in which they want, versus practitioners' and researchers' notions about ‘well‐considered’ decision‐making.^40^ Indeed, it is arguable that asking people with UM to review decisions *that* seem clear‐cut to them imposes an additional burden at a time of difficulty. Nonetheless, we are concerned that participants who agreed to accept a prognosis are exposing themselves to risks that they have not explicitly considered. At a population level, a poor prognosis is a more potent risk for distress than no prognosis.^10^ Thus, in seeking to know their prognoses, participants may increase the jeopardy of the distress that they want to avoid.

This said, any intervention that encourages greater consideration of risk should not imperil autonomy, meaning that interventions to prompt ‘well‐considered’ decisions should not seek to simply impose or insinuate practitioners' preferences either explicitly or implicitly.^20^ Preference *exploration* is a nondirective way of facilitating considered decision‐making, initially designed to enhance individuals' decisions about participation in clinical trials.^42^ It is designed to balance individuals' autonomy to make their own decisions with ‘well‐considered’ decision‐making. The guiding principles are acknowledging individuals' decisions as valid, but nondirectively encouraging and helping them to explicate and reflect upon their own reasonings.^51^, ^52^, ^53^ Preference exploration can lead to greater decision clarity, greater openness to previously overlooked considerations and more intensive consideration.^53^ It is notable that three participants changed their minds because they spontaneously engaged in preference exploration; revisiting decisions in light of procedural risk.

In UM, preference exploration could potentially address two key issues; first, that some participants did not fully understand its implications, and, second, that some did not consider relevant factors such as the possibility of a poor result. Preference exploration may also perform a symbolic function for those who do not intend to change their decisions. In our interviews, many *participants* developed plausible justifications for their decisions that they may not have otherwise done. Preference exploration encourages participants to justify their decisions. This may provide protection from postdecisional regret.^54^, ^55^

Many of our participants found that the process of understanding and making their decision evolved over time rather than a singular event.^18^, ^21^ Thus, preference exploration would need to be flexibly conducted as a single event or tailored to differing patient trajectories. Further, several participants experienced their prognostication decisions as stressful, and a framework for *concomitant* emotional support would need to be established.^56^

### 4.1 Limitations

Some limitations need to be borne in mind. Views from those who did not consent to testing may provide a more rounded picture. For example, their perceptions of uncertainty may be informative in understanding decision‐making. We did not have access to objective records of consultations in which prognostication was offered, and thus rely upon participants’ accounts of these. Although we took care to consider wider interpretations of contexts influencing decision‐making, the professional homogeneity of the analysis group could lead to a narrow band of interpretations based on individual psychology of decision‐making.

Participants were offered prognostication at a single unit that has offered prognoses for over 15 years.^10^, ^34^, ^42^ Transfer of the UM paradigm to other cancers needs to be handled carefully. Our *findings* should be seen as clarifying clinical and ethical issues in a context where decision‐making is not confounded by questionable accuracy or the prospect that a prognosis may lead to better treatments. These features are not always evident in prognostication dilemmas and our suggestions may need to be tempered.^57^ Similarly, we also caution that the majority of the sample is collected from a single unit located in a specific geographical area.

### 4.2 Conclusion

A single goal of *reducing* future worry associated with uncertainty drove participants' decisions to seek prognoses. While accepting the legitimacy of their wishes, their decisions seemed to reflect an incomplete consideration of the possibility of a poor prognosis. Preference exploration techniques may encourage people with UM to reflect upon this possibility.

## AUTHOR CONTRIBUTIONS

**Stephen L. Brown**: Conceptualization; data curation; formal analysis; funding acquisition; methodology; validation; visualization; writing – original draft. **Peter L. Fisher**: writing – review and editing. **Andrew Morgan**: Investigation; writing – review and editing. **Cari Davies**: Formal analysis; writing – review and editing. **Yasmin Olabi**: Formal analysis. **Laura Hope‐Stone**: Data curation; funding acquisition; investigation; resources; writing – review and editing. **Heinrich Heimann**: Funding acquisition; resources. **Rumana Hussain**: Resources; writing – review and editing. **Mary Gemma Cherry**: Formal analysis; methodology; validation; visualization; writing – review and editing.

## CONFLICTS OF INTEREST

The authors declare no conflicts of interest.

## Data Availability

The data that support the findings of this study are available on request from the corresponding author, but this provision may be subject to ethical review. The data are not freely available due to privacy or ethical restrictions.

## Footnotes

a It is possible, although uncommon, for people to agree to testing but to not immediately want the results. All patients in this study wanted prognostic estimates, thus for simplicity, we use the term prognostication to refer to the process of obtaining a tissue sample, testing that sample and the participant being informed of the results.
